# Whole Genome Sequencing and a New Bioinformatics Platform Allow for Rapid Gene Identification in *D. melanogaster* EMS Screens

**DOI:** 10.3390/biology1030766

**Published:** 2012-12-05

**Authors:** Michael A. Gonzalez, Derek Van Booven, William Hulme, Rick H. Ulloa, Rafael F. Acosta Lebrigio, Jeannette Osterloh, Mary Logan, Marc Freeman, Stephan Zuchner

**Affiliations:** 1 Dr. John T. Macdonald Foundation Department of Human Genetics and John P. Hussman Institute for Human Genomics, University of Miami Miller School of Medicine, Miami, FL, USA; E-Mails: mgonzalez15@med.miami.edu (M.A.G.); dvanbooven@med.miami.edu (D.V.B.); whulme@med.miami.edu (W.H.); rulloa@med.miami.edu (R.H.U.); racosta2@med.miami.edu (R.F.A.L.); 2 Department of Neurobiology University of Massachusetts Medical School, Howard Hughes Medical Institute, Worcester, MA, USA; E-Mails: jeannette.osterloh@umassmed.edu (J.O.); marc.freeman@umassmed.edu (M.F.); 3 Jungers Center for Neurosciences Research, Department of Neurology Oregon Health & Science University, Portland, OR, USA; E-Mail: loganm@ohsu.edu

**Keywords:** Next-generation sequencing, *Drosophila melanogaster*, EMS screen

## Abstract

Forward genetic screens in *Drosophila melanogaster* using ethyl methanesulfonate (EMS) mutagenesis are a powerful approach for identifying genes that modulate specific biological processes in an *in vivo* setting. The mapping of genes that contain randomly-induced point mutations has become more efficient in *Drosophila* thanks to the maturation and availability of many types of genetic tools. However, classic approaches to gene mapping are relatively slow and ultimately require extensive Sanger sequencing of candidate chromosomal loci. With the advent of new high-throughput sequencing techniques, it is increasingly efficient to directly re-sequence the whole genome of model organisms. This approach, in combination with traditional chromosomal mapping, has the potential to greatly simplify and accelerate mutation identification in mutants generated in EMS screens. Here we show that next-generation sequencing (NGS) is an accurate and efficient tool for high-throughput sequencing and mutation discovery in *Drosophila melanogaster.* As a test case, mutant strains of *Drosophila* that exhibited long-term survival of severed peripheral axons were identified in a forward EMS mutagenesis. All mutants were recessive and fell into a single lethal complementation group, which suggested that a single gene was responsible for the protective axon degenerative phenotype. Whole genome sequencing of these genomes identified the underlying gene *ect4*. To improve the process of genome wide mutation identification, we developed Genomes Management Application (GEM.app, https://genomics.med.miami.edu), a graphical online user interface to a custom query framework. Using a custom GEM.app query, we were able to identify that each mutant carried a unique non-sense mutation in the gene *ect4* (*dSarm*), which was recently shown by Osterloh *et al.* to be essential for the activation of axonal degeneration. Our results demonstrate the current advantages and limitations of NGS in *Drosophila* and we introduce GEM.app as a simple yet powerful genomics analysis tool for the *Drosophila* community. At a current cost of <$1,000 per genome, NGS should thus become a standard gene discovery tool in EMS induced genetic forward screens.

## 1. Introduction

*Drosophila melanogaster* is an extensively studied genetic model organism that has been used for the investigation of biological processes common to other organisms, including humans. Over the past century, sophisticated genetic approaches have been developed for *Drosophila* and the use of these methods have led to the illumination of fundamental principles in genetics and biology [1]. The availability of such techniques has made *Drosophila* one of the most powerful genetic model organisms. 

Forward genetic screens theoretically allow for the unbiased and exhaustive identification of sets of genes and genetic modifiers responsible for any phenotype of interest [2]. In particular, ethyl methanesulfonate (EMS) mutagenesis has been widely used to efficiently induce random point mutations. The advantages of EMS are a high rate of mutation, and production primarily of point mutations. Such mutations include strong loss of function changes, gain-of-function alleles, and DNA variants that result in the alteration of specific protein domains highly informative regarding function. However, a major obstacle of EMS-based mutagenesis is the subsequent identification of the causative lesions. In most cases, traditional genetic mapping has been used in *Drosophila* to identify small chromosomal loci linked to the phenotype of interest. The majority of these fine-mapping procedures are labor, cost, and time intensive. In addition, low recombination rates and insufficient mapping stocks hinder the ability to map mutations to some genomic regions. Finally, extensive Sanger sequencing of regions of interest are generally required to successfully identify the precise causative lesion. 

The development of next-generation sequencing (NGS) has dramatically improved the throughput and cost of sequencing. The size of the *Drosophila* genome is ~180 million bases (Mb) and is predicted to contain 13,601 protein coding genes [3]. The current reference genome dm3 is based on the Berkeley Drosophila Genome Project (BDGP) and reliable annotation is available *via* Flybase, ENSEMBL, UCSC Genome Browser and others. Information on common and rare natural variation in the *Drosophila* genome is less well curated, but available from various sources (http://flybase.org, http://dpgp.org). Currently, a whole *Drosophila* genome can be sequenced within a few weeks for <$1,000, but this price point will likely drop rapidly in coming years. Not surprisingly, a small number of studies have begun to explore the possibility of applying NGS to EMS-induced gene identification. These studies have shown in principle that a known mutation can be re-identified [4], and that sequence capture/enrichment of regions of interest will also allow for reliable mutation detection [5]. Albeit the technical challenges of NGS appear largely resolved, user-friendly bioinformatics analysis tools designed for the wet lab based molecular-oriented *Drosophila* scientist are still warranted. Each *Drosophila* genome represents a large amount of data and can thus be challenging to analyze. However, the main routes of analysis for the purpose of gene mapping can be summarized in a standardized bioinformatics pipeline. Here we present a novel genomes management tool with a graphical user interface that is accessible through any major web browser. In order to demonstrate the effectiveness of NGS in the identification of causative mutations, we present detailed data from a recent discovery of the first natural gene required for Wallerian degeneration [6]. 

## 2. Methods

### 2.1. Sample Preparation

For DNA preparation, 200 anesthetized flies were flash frozen on dry ice then homogenized in 300 μL of sterile-filtered Buffer A (10 mM Tris-Hcl, pH7.5; 60 mM NaCl; 10 mM EDTA; 150 nM spermine; 150 nM spermidine; 5% sucrose; 0.5% Triton-X). After centrifuging at 4C at 15,000 RPM for 15 minutes, the supernatant was discarded and the nuclei (pasty layer of the pellet) was carefully resuspended in 300 μL l Buffer A. The resuspended nuclei were transferred to a tube containing 300 μL of sterile-filtered Buffer B (2% N-lauryl sarcosine; 0.1M EDTA; 5% sucrose) and heated to 65 °C for 45 minutes. 800 μL of 1:1 phenol:chloroform mix (Fisher Scientific) was added to the mixture followed by vortexing for 10 seconds. After a 10 second rest, the mixture was vortexed again for 10 seconds and then spun at 15,000 RPM for 5 minutes at room temperature. The supernatant was removed and mixed with 600 μL chloroform. The mixture was vortexed and spun as before. The supernatant was removed and an equal volume of isopropanol was added. After several inversions, the mixture was spun at 4 °C at 15,000 RPM for 15 minutes. The pellet was then washed with 500 μL of 70% ethanol and spun at 4 °C at 15,000 RPM for 5minutes. The supernatant was removed and the pellet was allowed to air dry before resuspension in TE2 with RNAse (10 mM Tris-Hcl, pH7–8; 2mM EDTA; 10 ug RNAse A). The mixture was incubated at 37 °C for 30 minutes. The DNA was reprecipitated with 200 μL 100% ethanol and then spun at 15,000 rpm for 15 minutes at 4 °C. The pellet was washed and resuspened as before with 70% ethanol. After air drying, the pellet was resuspended in TE2 (without RNAse). All chemicals are from Sigma unless otherwise noted.

### 2.2. Illumina Whole-genome Sequencing

Library construction of genomic DNA was performed according to the Illumina ® TruSeq™ DNA Sample Preparation protocol, including a gel-based size-selection such that final median insert size was 300 bp. Samples were barcoded to allow for multiplexing. Cluster generation took place on the Illumina cBot according to the manufacturer's recommendations. Sequencing was performed on the Illumina HiSeq2000 using the reagents provided in the Illumina TruSeq PE Cluster Kit v3 and the TruSeq SBS Kit – HS (200 cycle) kit. 

### 2.3. Data Analysis and Variant Detection

The Illumina CASAVA v1.4 and v1.5 pipeline was used to produce 74bp sequence reads. BWA [14] was used to align sequence reads to the *Drosophila melanogaster* reference genome (dm3) and variants were called using the GATK software package v1.4 [11]. All variants were submitted to ENSEMBL Variant Effect Predictor for further characterization. Genomes Management Application (GEM.app), University of Miami Miller School of Medicine (https://genomics.med.miami.edu) was utilized for analysis of variants detected. In order to reduce the number of false positive genotype calls, we used two of GATK Unified Genotyper’s quality scores to filter out low quality variations. GATK’s *genotype quality* score (GQ) is a Phred-scaled likelihood confidence score that the genotype of a variant is correct. For heterozygous variations, the GQ equation is as follows:


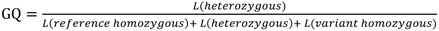
 with a max score of 99. The *QUAL* score is the Phred probability that a variant polymorphism exists at a given site given the sequencing data, where a score of 10 indicates a 1 in 10 chance of error and a score of 100 indicates a 1 in 100 chance of error.

## 3. Results

### 3.1. Whole Genome Sequencing Analysis of D. melanogaster

The Illumina HiSeq2000 sequencing platform was used to generate whole genome data sets on four *Drosophila* samples, a background strain (*y,w*) and three mutant strains (*mut1*, *mut2*, *mut3*). The mutant strains were identified in an EMS mutagenesis as capable of blocking axonal degeneration after axotomy. Each of these lines were homozygous lethal and we therefore performed whole genome sequencing on heterozygous animals crossed over the original background chromosome (e.g., *y,w* ; *mut1*/+). The sequence data corresponded to an average of ~74 million 74bp sequence reads per sample *(*control: 42,278,410; *mut1*: 83,425,870; *mut2*: 43,972,798; *mut3*: 125,284,692). Applying standard sequence read alignment software (BWA), 88% of paired-end reads were aligned to the *Drosophila* reference sequence (dm3) (Table 1). The resulting average sequence read depth per sample was 41X across the genome (Table 1). This coverage depth has been shown previously to be sufficient for the identification of heterozygous mutations [7]. EMS mutagenesis is known to induce point mutations by nucleotide substitution, particularly by guanine alkylation [8]. Therefore we limited our analysis to single nucleotide variations (SNVs). We also explored the efficiency of filtering for G/C to A/T transitions as 70%–100% of EMS induced mutations have been reported to fall in this category [9,10]. By applying the GATK software, we detected a total of 2,155,300 SNVs in three mutant strains, an average of ~718,000 variations per mutant genome (Table 1). Since many of the variant calls have very low quality or low sequence coverage we applied conservative quality filters. A GQ score of ≥ 75 and QUAL score ≥ 100 were required, which indicate a ≥99% chance of a variant at this site and 10^7.5^ likelihood that the genotype call is correct [11]. Application of these quality filters reduced the number of SNVs to ~390,000 per mutant, which were then considered for subsequent analyses (Table 1). 

**Table 1:** Whole genome sequencing metrics per sample.

Sample	# of reads	# of reads aligned	% reads aligned	Avg depth	# of SNVs	# of high quality SNVs	# of NS/SS cSNV	# of NS/SS cSNV on 3L	Unique NS/SS cSNV on 3L
Background	42,278,410	37,650,186	88.1	27.1	412,362	88,593	21,745	2,851	25
Mutant 1	83,425,870	72,784,178	87.2	44.6	849,658	456,778	35,172	6,544	863
Mutant 2	43,972,798	38,172,143	86.81	23.4	647,655	427,332	25,054	5,252	533
Mutant 3	125,284,692	111,178,422	88.7	68.1	657,987	231,449	32,114	4,913	48
Total in Mutants					2,155,300	1,115,559	92,340	16,709	1,444

# - number; SNV – single nucleotide variant; cSNV – coding SNV; NS/SS – nonsynonymous/splice site; 3L – chromosome 3L.

**Figure 1:**
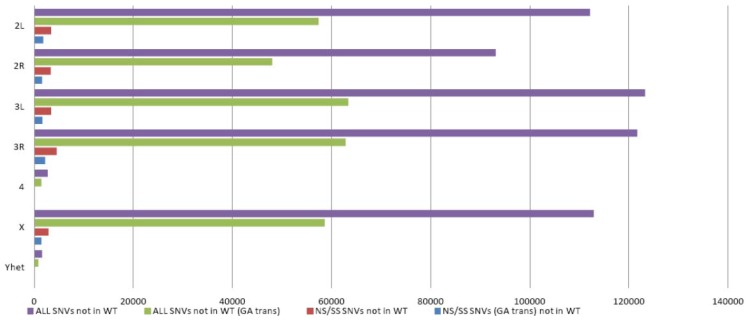
Distribution of detected SNVs not present in background on each chromosome.Purple bars represent the total number of SNVs that were observed in mutant genomes but not in the background genome. Green bars represent the number of SNVs that were G>A or C>T transitions. Red bars represent the number of nonsynonymous or splice-site SNVs that were not present in the wild-type genome and detected in the mutant genomes. Blue bars represent the number of nonsynonymous and splice-site SNVs that were G>A or C>T transitions.

To obtain a better understanding of the frequency of natural DNA variation and EMS induced mutations, we analyzed the distribution of SNVs per chromosome. The larger autosomes and the X chromosome contained between ~8,100 to ~11,000 homozygous and heterozygous variants per megabase (Mbp) of DNA sequence (Table 2). Chromosome 4 and YHet displayed less variant density (Figure 1). Approximately 3% of SNVs not shared between any mutant and background genomes were non-synonymous or splice-site variations (Figure 1). Of those, 52% represented G->A or C->T changes, in line with the EMS induced guanine alkylation. This number is at the lower end of previous observations [9,10], but is likely explained by us only analyzing one parental genome. Overall we observed a mutation rate of ~0.6 mutations per 1kb, which coincides with previous reports [5]. 

**Table 2:** Observed number of SNVs per million base pair (Mbp) in each chromosome.

Chromsome	Variants per Mbp
2L	11023
2R	9848
3L	11043
3R	8554
4	3331
X	8108
Y	72

### 3.2. Gene Identification Applying WGS

**Figure 2:**
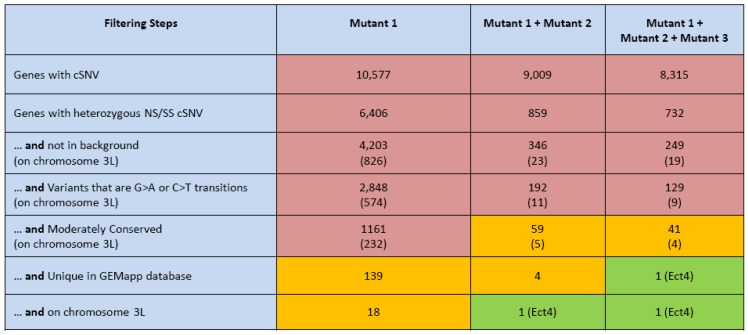
Filtering strategies used for gene identification by whole genome sequencing. Each box lists the number of genes with one or more protein-coding SNV (cSNV). Columns show the effect of requiring that one or more cSNV be observed in each of one to three mutant flies. Rows show the effect of filtering variants that meet certain analysis criteria. Row 6 displays the effect of including 8 samples from different screens in analysis.

We sequenced genomic DNA from a background strain and three heterozygous non-complementing mutants. The phenotype of these mutants was delayed axonal degeneration upon nerve severance and the responsible gene and its biology was recently published [6]. Beginning with the entire number of homozygous and heterozygous changes in each of the four genomes, we devised a step-wise filtering approach that would reduce the number of SNVs in each individual mutant strain. Cross-comparisons between the non-complementing strains would then possibly identify the underlying gene. Firstly, we limited analysis to the ~10,000 genes per mutant strain that contained a coding variant (Figure 2). When only considering heterozygous non-synonymous and splice-site changes the number of genes was reduced by ~40%. Further, the variant should not be present in the background strain (reduction to ~4,000 genes) and should be an EMS induced G>A or C>T transition (reduction to ~2,900 genes) (Figure 2). Next, we used phylogenetic analysis based conservation scores (*PhastCons*) of 15 fly species available on the UCSC genome browser (http://genome.ucsc.edu/). *PhastCons* predictions are based on a phylogenetic hidden Markov model to identify conserved elements in multiple aligned sequences [12]. Using these scores, we filtered for moderately conserved variants (PhastCons > 0.5) further reducing the number to 1161 candidate genes (Figure 2). In addition, it is unlikely that the same mutation will occur in eight other *Drosophila* genomes that we have sequenced for other unrelated projects (reduction to 139 genes across the whole genome). Finally, since the initial screen was performed on chromosome 3L, we were able to reduce the number of candidate genes to 18 (Figure 2). 

**Figure 3:**
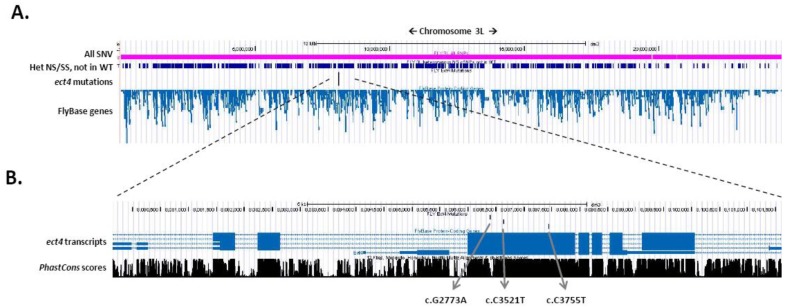
Mutation density on chromosome 3L in screen (N = 3). (A) Screenshot of chromosome 3L with custom tracks in the UCSC genome browser. The pink line displays all SNVs on chromosome 3L that were detected in this screen. The navy line displays all heterozygous non-synonymous and splice-site SNVs on chromosome 3L not present in the wild-type fly. (B) Screen shot of *ect4/dSarm* with custom track displaying the location and conservation of the three *ect4* mutations.

The power to detect the underlying gene dramatically increases if multiple non-complementing strains are available suggesting that each strain has a unique mutation in the same gene. When combining the data from two such strains the number of possible genes in the whole genome is reduced to four and only one gene, *ect4*, contained significant unique changes in each mutant strain on chromosome 3L (Figure 2). As expected, by adding a third non-complementing strain, *ect4* can be directly identified genome wide (Figure 2 and Figure 3).

The proposed filtering strategy will differ depending on investigator opinions for appropriate cut-offs for conservation, the order of filter options, the availability of non-complementing mutant strains, and the extent of available genetic mapping data. Ideally, a software application will allow for a rapid and iterative trial and error approach towards optimizing the filtering strategy for each screen and dataset. We have therefore developed a user-friendly web-based tool, which can be tailored to fit the needs of individual projects and users to analyze and compare entire *Drosophila* genomes within seconds.

### 3.3. The Genomes Management Application (GEM.app)—A Novel Tool for Rapid Genome Analysis and Comparison

**Figure 4:**
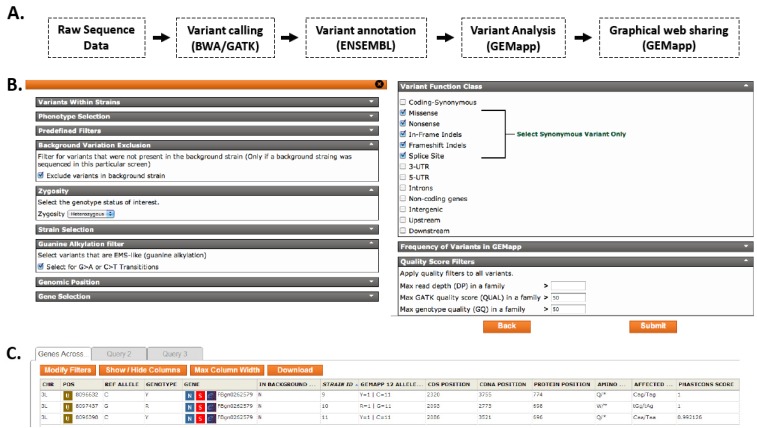
GEM.app bioinformatics pipeline and online graphical interface. (A) The systematic processes of the automated GEM.app bioinformatics pipeline. (B) Screenshot of the GEM.app form with some of the filtering options displayed. (C) The output of GEM.app query used to identify *ect4* mutations. A subset of 34 total columns are shown. Links within the table (U, N, S, e) provide direct access to common resources, such as UCSC genome browser, NCBI, String gene networks, and Ensembl. Up to three tabs allow for parallel queries. Additional options include modifying filter settings, managing columns, and downloading results in an excel sheet.

In order to facilitate the storage, annotation, analysis, and visualization of NGS variant data, we have developed Genomes Management Application (GEM.app, https://genomics.med.miami.edu), an online graphical interface to a custom query framework. GEM.app imports data from a standardized and automated variant calling pipeline; in our case a BWA/GATK pipeline that produces files in the variant call format (VCF) (see methods for details). The genome data import module (GEDI) of GEM.app handles the annotation of all variants, the calculation of frequency counts across samples in a central database, and also determines for each variant if the original VCF files contain the reference allele or reports missing data (“backfilling”) (Figure 4a). A graphical user interface eliminates the need for command line or bioinformatics knowledge (Figure 4b). For data queries, GEM.app is accessed in a standard web browser and allows for the selection of multiple filters, which largely replicate the analytical strategy described above. Specifically, the user can filter for variant function class (synonymous, nonsynonymous, nonsense, *etc.*), zygosity (homozygous, heterozygous, hemizygous), allele frequency of variants across the entire database, conservation scores, chromosomal regions and genes of interest, and guanine alkylation derived changes. Most importantly, analysis across samples in a project is possible and allows utilizing the power of multiple non-complementing strains. After submission of query criteria, output is displayed in the web browser, which includes features such as direct links to ENSEMBL gene viewer, a gene network viewer [13], and the optional download of results in a tab-delimited format that directly opens in Excel (Figure 4c). Benchmarking of query times revealed an average of 0.15 seconds for a single whole *Drosophila* genome. Queries across four whole genomes as presented in this paper averaged 0.27 seconds. 

In addition to being user-friendly and fast, GEM.app is a centralized data storage system for many studies. GEM.app is password protected and each user has restricted rights only to their own genome data. It is very convenient for a single laboratory and its multiple lab members to access genomic data from any computer or mobile device. In addition, every new sample that is added improves the knowledge on variant frequencies in the *Drosophila* genome and these frequencies are available to all users in an anonymous count. For example, when we added the genomic information from eight additional EMS-induced *Drosophila* genomes not related to the presented study, we were able to reduce the number of remaining genes by ~90% (Figure 2). 

## 4. Discussion

Before the advent of whole genome sequencing, fine mapping techniques were used to identify mutations involved in phenotypes of interest in genetic screens followed by Sanger sequencing. This approach has been very successful, but can be time-consuming and expensive. Sequencing and analysis of the whole *Drosophila* genome now takes 2-3 weeks and costs <$1,000, making NGS an increasingly inexpensive and potentially high-throughput experiment. In our experience, a full Illumina HiSeq2000 run could produce 64 high-quality *Drosophila* genomes. Further price reductions will likely stimulate large-scale EMS screens, because it allows for an unbiased search for mutants across whole genomes. The biggest conceived obstacle to a wider adoption of this approach is arguably the bioinformatic challenges posed by whole genome analysis. More specifically, the user-friendly accessibility of genome data to the molecular-genetic-oriented investigator is essential for the widespread implementation of these techniques. 

Our results demonstrate that whole genome sequencing is an effective method to identify EMS-induced variants in a novel gene underlying a *Drosophila* model for delayed Wallerian degeneration [6]). This is in line with other studies that have applied targeted NGS and whole genome sequencing to *Drosophila* [4,7]. We showed that a number of experimental and analytical strategies are viable:

1. If a single mutant line exists, the parallel sequencing of the background strain and mapping to a chromosomal arm will reduce the number of genes carrying a strong coding variant to less than 20, possibly even identifying a strong mutation outright.
2. It is most advantageous to add a second non-complementing strain to filter for genes that contain strong variants in both strains. In this case, further mapping of the gene is not even necessary in most cases (Figure 2).
3. Adding more *Drosophila* genomes, related or unrelated to a project, has a great potential to further eliminate variants with a low frequency in the *Drosophila* population; or alternatively, nucleotide positions that are frequently hit by the EMS mutagen.

In our opinion, this approach can be further improved by utilizing evolutionary conservation scores and, in the future, by adding comprehensive *in silico* predictions for protein changes as they exist in human genetic maps. 

It is important to mention, that this approach has some potential shortcomings, which will not always lead to straight forward mutation identification. First, we have focused analysis on coding variation. If the underlying mutation is in non-coding, intronic, or intergenic regions the number of potential variants is much larger. This could, however, be addressed by positional fine mapping. Second, repetitive or homologous regions of genomes are notoriously difficult to cover with short-read sequences that are produced by the major NGS platforms. Finally, current bioinformatics techniques and NGS sequencing protocols require specialized approaches to detect large chromosomal structural variations, large insertions/deletions, or copy-number variations. GEM.app does not currently provide these analyses.

Analyzing and visualizing variant data can be extremely daunting to investigators with limited computational experience. In order to address the bioinformatics challenge we are presenting the Genomes Management Application (GEM.app). The framework of GEM.app has been developed for human, mouse, and fly genomic data, but we will describe the human and mouse versions in separate papers. GEM.app provides a graphical online user interface, allowing users of varying computational skills to analyze NGS data. Little to no prior experience is required to filter existing data and download the results to a local computer. In addition, GEM.app is an application that allows for centralized data storage, annotation, and analysis. An advantage of this approach is the accumulation of variant frequencies across different projects and users. Each user has a unique account with access to their data only. However, anonymous variant frequency counts are available to all users across GEM.app. The currently available queries are designed to meet most needs for the filtering of EMS-induced variants in single strains or across non-complementing strains. A great advantage of GEM.app is the speed of analysis, which enables a bioinformatics naïve investigator to test different filter strategies or varying cut-offs exhaustively and obtain immediate feedback. The identification of *ect4* as described above is now literally achieved in seconds. 

## 5. Conclusions

Connecting the efficiency of EMS mutagenesis with the high-throughput capabilities of NGS and a powerful yet simple analysis approach will undoubtedly speed up the characterization of genes. As the number of available *Drosophila* genomes increases, we will rapidly refine our understanding of the effect of EMS mutations on gene and protein function and the roles they play in mutant phenotypes.
